# Leading the charge in the education sector: development and validation of the School Implementation Leadership Scale (SILS)

**DOI:** 10.1186/s13012-022-01222-7

**Published:** 2022-07-19

**Authors:** Aaron R. Lyon, Catherine M. Corbin, Eric C. Brown, Mark G. Ehrhart, Jill Locke, Chayna Davis, Elissa Picozzi, Gregory A. Aarons, Clayton R. Cook

**Affiliations:** 1 Department of Psychiatry and Behavioral Sciences, University of Washington, 6200 NE 74th Street, Suite 100, Seattle, WA 98115 USA; 2 Department of Public Health Sciences, Miller School of Medicine, University of Miami, 1120 NW 14th Street, Office, 104, Miami, FL 33136 USA; 3 Department of Psychology, University of Central Florida, P.O. Box 161390, Orlando, FL 32816-1390 USA; 4 Department of Psychiatry, University of California, San Diego, USA; 5 UC San Diego ACTRI Dissemination and Implementation Science Center, San Diego, USA; 6 Child and Adolescent Services Research Center, San Diego, USA; 7 Department of Organizational Leadership and Policy Development, University of Minnesota, 206 Burton Hall-178 Pillsbury Drive SE, Minneapolis, MN 55455 USA

**Keywords:** Implementation leadership, Measurement, Education, Prevention, Mental health

## Abstract

**Background:**

Strategic implementation leadership is a critical determinant of successful implementation, hypothesized to create a more supportive implementation climate conducive to the adoption and use of evidence-based practices. Implementation leadership behaviors may vary significantly across contexts, necessitating studies that examine the validity of established measurement tools in novel health service delivery sectors. The education sector is the most common site for delivering mental health services to children and adolescents in the USA, but research focused on implementation leadership in schools is in the early phases, and there is a need for adaptation and expansion of instruments in order to tailor to the school context. The current study adapted and validated the School Implementation Leadership Scale (SILS) (based on the Implementation Leadership Scale) in a sample of elementary school personnel from six school districts who were implementing one of two well-established prevention programs for supporting children’s mental health.

**Methods:**

Participants were 441 public school teachers from 52 elementary schools in the Midwest and West Coast of the USA. Participants completed a survey that contained: (1) an adapted and expanded version of the SILS with additional items generated for four existing subscales as well as three new subscales (communication, vision/mission, and availability), and (2) additional tools to evaluate convergent and divergent validity (i.e., measures of general/molar leadership and teaching attitudes). Data underwent (1) examination of item characteristic curves to reduce items and ensure a pragmatic instrument, (2) confirmatory factor analyses to establish structural validity, and (3) evaluation of convergent and divergent validity.

**Results:**

Item reduction analyses resulted in seven subscales of three items each. Results indicated acceptable fit for a seven-factor structural model (CFI = .995, TLI = .99, RMSEA = .07, SRMR = 0.02). Second-order factor loadings were high (λ = .89 to .96), suggesting that the SILS subscales comprise a higher-order implementation leadership factor. All subscales demonstrated good inter-item reliability (α = .91–.96). Convergent and divergent validity results were generally as hypothesized, with moderate to high correlations between SILS subscales and general leadership, moderate correlations with teaching attitudes, and low correlations with school demographics.

**Conclusions:**

Overall, results provided strong structural, convergent, and divergent validity evidence for the 21-item, 7-factor SILS instrument. Implications for the measurement of implementation leadership in schools are discussed, as well as strategies to support leaders to enhance their strategic behaviors related to the implementation of mental health prevention programs (e.g., adaptation of existing leadership-focused implementation strategies).

**Supplementary Information:**

The online version contains supplementary material available at 10.1186/s13012-022-01222-7.

Contributions to the literature

- The education sector is the most common location for the delivery of mental health services for children and a setting where organizational leadership is critical to the implementation of evidence-based practices, but few instruments exist to evaluate implementation leadership behaviors.
- Based on the Implementation Leadership Scale, the current study adapted and validated the School Implementation Leadership Scale for use by educators implementing evidence-based prevention programs to promote children’s mental health.
- Findings revealed an expanded seven-factor School Implementation Leadership Scale that demonstrated a structure similar to the original Implementation Leadership Scale as well as convergent and divergent validity evidence with related and unrelated instruments, respectively.

## Introduction

Measurement of organizational influences on successful implementation of evidence-based practices (EBP) is a clear priority for implementation research, given consistent findings regarding their impact on implementation outcomes [1–3]. Organizational structures and processes—such as leadership behaviors—can vary widely across contexts, necessitating studies that examine the application and validity of established measurement tools in novel health service delivery sectors. Among these, the education sector is the most common site for delivering prevention and intervention services targeting child and adolescent mental health [4]. However, EBP are inconsistently adopted, delivered with fidelity, or sustained over time in schools [5, 6]. Barriers related to support from school leadership are commonly cited as reasons why EBP implementation fails in the school setting [7, 8]. Despite the availability of a psychometrically sound measure of implementation leadership (Implementation Leadership Scale [ILS]) [9] in other health and mental health delivery contexts, no research has systematically validated an instrument to assess strategic implementation leadership in schools.

### Organizational leadership for implementation

Research suggests that the inner context—characteristics of the immediate organizational setting in which implementation occurs [10, 11]—is critical to the successful implementation of EBP [12–16]. As a key driver of inner context organizational functioning, the skills and behaviors of leaders are frequently highlighted across organizational types, including in schools [17–19]. Research on organizational leadership can take a *general* (i.e., molar) approach, indicating the typical behaviors performed when interacting with their subordinates (e.g., transformational leadership [20]), or a *strategic* approach, focusing specifically on those leadership behaviors that support or inhibit a strategic goal (such as customer service [21] or safety [22]). Leaders who accomplish their strategic goals engage in routine interactions and communications with staff that support those goals, protect time during meetings to discuss related content, hold staff accountable, and provide performance-based feedback related to strategic goals [23, 24]. Meta-analytic findings indicate that such approaches are useful and often have strong relationships with strategic climates (e.g., service climate) and quality outcomes, such as customer satisfaction and financial outcomes [25], because of their focus on the specific aspects of leadership that are most relevant to and predictive of particular outcomes of interest (e.g., high-quality customer service).

Within implementation research, strategic implementation leadership has been hypothesized to be a critical precursor of strategic implementation climate [26] and to be more directly related to implementation outcomes (e.g., feasibility, fidelity, reach) than general/molar leadership. Strategic implementation leadership is made up of behaviors (e.g., strategic communication and direct support) that serve an embedding function (i.e., what leaders do to achieve a strategic objective) for new practices and programs [27]. Rigorous assessment of implementation leadership is critical to inform implementation research and practice in this domain as recent studies have shown that implementation leadership is a malleable organizational characteristic [28]. Aarons, Ehrhart, and Farahnak [9] developed the Implementation Leadership Scale (ILS) to capture strategic leadership behaviors that drive successful EBP implementation. Designed to be brief and pragmatic [29], the original ILS contained 12 items loading onto 4 subscales: Proactive Leadership (anticipating and addressing implementation challenges), Knowledgeable Leadership (deep understanding of EBP and implementation issues), Supportive Leadership (support for EBP adoption/use), and Perseverant Leadership (consistent and responsive to challenges). Subscale internal consistencies ranged from 0.95 to 0.98. Although the ILS was originally developed in specialty mental health, the authors called for research examining its utility in other service settings [9]. Subsequent studies have begun this work, including successful validation of the instrument in child welfare [30], substance use treatment [31], and acute care [32]; as well as translation into other languages (e.g., [33, 34]) and adaptation to measure sustainment-focused leadership [35]. The ILS has recently been identified as one of only a few leadership instruments with adequate evidence for its use in healthcare [36].

### School-based mental health services

A wealth of research has highlighted the central role that the education sector plays in mental health services for children and adolescents. Internationally, school-based mental health programs have grown markedly over the past two decades [37–40]. In the USA, studies consistently indicate that the education sector is the most heavily accessed youth mental health service setting, responsible for 50–80% of all mental healthcare [4, 41–45]. Increasingly, school-based mental health programs are organized via a multi-tiered system of support (MTSS) framework. MTSS is grounded in the public health model of prevention and conceptualized as a three-tier framework that provides a data-driven continuum of intensifying supports [46, 47]. The foundation of MTSS is the universal level in which EBPs are delivered to all students to prevent the emergence of student mental health problems [48, 49]. Unfortunately, despite strong evidence for a wide variety of universal prevention programs for student mental health, findings suggest that inconsistent implementation is common and that problems with delivery attenuate their impact [50–53]. Organizational leadership is a critical determinant of the successful implementation of these programs.

### Implementation leadership in schools

While implementation research in schools has recently increased, the contributions of leadership and other organizational influences on successful implementation of EBPs in that context remain understudied [6]. Although leadership models vary widely across schools [54, 55], all schools have a site-based principal or headmaster who has the authority to make decisions and hold staff accountable for achieving implementation objectives. Prior organizational research in the education sector has focused on principal managerial or instructional leadership [56] and has shown positive leadership to increase staff productivity [57], and established its links to school climate and student outcomes [58]. Related to mental health, research has established that principal buy-in and school leadership are critically important to the implementation of universal prevention programs [58–61]. Other studies are examining leadership in relation to implementation of interventions for autism spectrum disorders [8, 17, 18, 62, 63] and characterizing leadership styles conducive to implementation [19].

Despite strong interest, research focused on implementation leadership in schools has been significantly hampered by instrumentation limitations. Existing measures of principal leadership (e.g., [64, 65]) tend to assess global leadership qualities and are too broad to track the specific leadership behaviors most associated with the adoption, high-fidelity use, and sustainment of EBPs. Many specific programs have developed their own “readiness” assessments, but they are neither specific to leadership nor generalizable across EBPs. Pragmatic and brief instruments are needed to assess implementation leadership in schools. In pursuit of this objective, Lyon and colleagues conducted an initial adaptation of the ILS for use with mental and behavioral health consultants in the education sector [66]. These consultants functioned primarily as implementation intermediaries to support the installation of school-based programs. Items underwent minor surface-level adaptations (e.g., replacing “agency” with “school,” “clinician” with “school personnel,” etc.), but efforts were made to preserve the integrity of the original items and constructs. Findings provided strong support for the original ILS factor structure with this new population. However, because it was administered to implementation consultants/intermediaries, the instrument was not designed for teachers, the primary deliverers of mental health prevention programming in schools. It also did not include the full range of relevant school leader behaviors that can support implementation. As a result, the ILS was further revised through a series of focus groups with educators (central administrators, principals, teachers [67];) and pilot tests to understand the applicability and conceptual boundaries of the concept of implementation leadership and ensure the instrument’s alignment with the realities of educational settings implementing universal prevention programs. This process produced an initial *School Implementation Leadership Scale* (SILS, see “Method” section), which was refined and tested in the current study.

### Study aims

In light of the need for reliable, valid, and pragmatic instruments to measure strategic implementation leadership in the education sector, the current study administered an adapted SILS to teachers who were implementing one of two different universal EBPs (Schoolwide Positive Behavioral Interventions and Supports (SWPBIS [68];) and Promoting Alternative Thinking Strategies (PATHS [69];) across six elementary school districts. The evidence for universal prevention programming in mental health is strongest at the elementary level, making elementary schools a priority for increasing the public health impact of evidence-based mental health prevention practices. The resulting data were used to (1) conduct item reduction analyses to ensure a pragmatic instrument [29], (2) complete a confirmatory factor analysis to establish structural validity, and (3) evaluate convergent and divergent validity with measures of molar leadership, staff attitudes, and school demographic variables. We hypothesized that the SILS would demonstrate moderate to high associations with molar leadership (convergent and divergent validity) and lower associations with attitudes and demographics (divergent validity).

## Method

### Setting and participants

#### Setting

Schools implementing one of two evidence-based interventions (*n* = 39 SW-PBIS; *n* = 13 PATHS) were eligible and recruited for participation, resulting in 441 teachers from 52 elementary schools in 6 school districts in Washington, Ohio, and Illinois. The average racial/ethnic and socioeconomic composition of students across schools was 66% Non-White (range 21 to 100%) and 57% low-income status (range 4 to 100%), respectively. Across all participating schools, an average of 88% of teachers who were contacted to participate (*n* = 500) completed the study’s online survey (see “Procedures” section below).

#### Teacher-level demographics

On average, 9 teachers per school were recruited to complete measures. Most teachers were female, had at least a master’s degree, had an average of 11.6 years of experience, and were predominately White (see Table 1 for complete demographic information). The number of participants included in analyses was sometimes less than 441 due to missing data (< 5% overall).

**Table 1:** *P*articipant demographics for School Implementation Leadership Scale (SILS) general (*N* = 219), specific (*N* = 222), and combined (*N* = 441) samples

**Participant information**	**General** **Freq (%)**	**Specific** **Freq (%)**	**Combined** **Freq (%)**
Age			
18 to 24 years old	7 (3.2)	14 (6.3)	21 (4.8)
25 to 34 years old	65 (29.8)	64 (29.0)	129 (29.4)
35 to 44 years old	58 (26.6)	63 (28.5)	121 (27.6)
45 to 54 years old	56 (25.7)	47 (21.3)	103 (23.5)
55 to 64 years old	31 (14.2)	30 (13.6)	61 (13.9)
65 to 74 years old	1 (0.5)	3 (1.4)	4 (0.9)
Total	218 (100.0)	221 (100.0)	439 (100.0)
Gender			
Male	27 (12.4)	19 (8.6)	46 (10.5)
Female	190 (87.2)	201 (91.4)	391 (89.3)
Other	1 (0.5)	0 (0.0)	1 (0.2)
Total	218 (100.0)	220 (100.0)	438 (100.0)
Race			
American Indian or Alaskan Native	7 (3.2)	1 (0.5)	8 (1.8)
Asian	1 (0.5)	5 (2.3)	6 (1.4)
Black or African American	14 (6.5)	8 (3.7)	22 (5.1)
Native Hawaiian or Pacific Islander	0 (0.0)	1 (0.5)	1 (0.2)
White or Caucasian	179 (82.5)	184 (85.2)	363 (83.8)
Multiracial	11 (5.1)	10 (4.6)	21 (4.8)
Other	5 (2.3)	7 (3.2)	12 (2.8)
Total	217 (100.0)	216 (100.0)	433 (100.0)
Ethnicity			
Latino/Hispanic	14 (6.4)	17 (7.7)	31 (7.1)
Non-Latino/Hispanic	204 (93.6)	203 (92.3)	407 (92.9)
Total	218 (100.0)	220 (100.0)	438 (100.0)
Highest degree earned			
Bachelors	72 (33.0)	68 (30.9)	140 (32.0)
Masters	145 (66.5)	152 (69.1)	297 (67.8)
Doctoral	1 (0.5)	0 (0.0)	1 (0.2)
Total	218 (100.0)	220 (100.0)	438 (100.0)
Grade			
K–2nd	92 (42.0)	99 (44.6)	191 (43.3)
3rd–5th and other	127 (58.0)	123 (55.4)	250 (56.7)
Total	219 (100.0)	222 (100.0)	441 (100.0)
	**PBIS T1** ***N*, Mean ± sd**	**PATHS** **N, Mean ± sd**	**COMBINED** **N, Mean ± sd**
Years in current role	218, 11.9 ± 6.9	220, 11.3 ± 7.1	438, 11.6 ± 7.0
Years at current school	218, 7.0 ± 6.1	220, 6.9 ± 5.9	438, 6.9 ± 6.0

#### Procedures

This study was part of a large-scale, federally funded measure adaptation and development project with the aim of creating school-adapted organizational assessments. Prior to conducting the current study, the original SILS was adapted for use in schools through (1) input from research and practice experts during a structured in-person convening and (2) mixed-methods focus group sessions with key educator stakeholder groups (central district administrators, principals, teachers) [67]. Adaptations included changing item wording to ensure construct equivalence for the target respondents (i.e., school-based practitioners) and deleting or expanding items and item content to ensure contextual appropriateness to schools [70]. An effort was made to preserve the integrity of the original items and constructs as much as possible [71]. Expansion included developing items to address additional constructs in subscales focused on leaders’ (a) communication, (b) organizational vision/mission, and (c) availability to support EBP implementation.

Human subjects’ approval was obtained from the University of Washington Institutional Review Board and participating school districts’ research and evaluation departments, when applicable. Study investigators first reached out to school district central administrators to discuss the project and secure participation. School recruitment was done in collaboration with central administrators who identified eligible school buildings and facilitated the distribution of information surrounding project benefits and data collection procedures to site-based administrators. Teachers from each school (*n* = 4–10) were then recruited by school administrators or a site-based liaison who typically presented the opportunity either during standing staff meetings or via email communications. Contact information for interested teachers was provided to research staff and used to establish and maintain project communications (e.g., sending survey links).

Data were collected between September and November of the 2017 academic year. In November, teachers were sent an initial email to provide a project overview, obtain informed consent, and provide a link to the online survey. Upon receiving the initial email, teachers had one-month to complete the online survey. Weekly email reminders were sent to increase the response rates at each school.

### Measures

#### School Implementation Leadership Scales (SILS)

The original ILS [9] and original SILS adaptation [66] are 12-item instruments developed to assess strategic leadership for EBP implementation. All ILS items are scored on a four-point Likert scale ranging from 0 (“not at all”) to 4 (“to a very great extent”). Both versions have previously supported a factor structure with four first-order factors (proactive leadership, knowledgeable leadership, supportive leadership, perseverant leadership)—each with three items—loading onto an overarching implementation leadership latent factor [26, 66]. As described above, the present study adapted the original SILS based on expert feedback, adding items for three new subscales (communication, vision, available). Eighteen additional items were initially developed for the new subscales and to augment the existing subscales with contextually appropriate items. This resulted in an initial 30-item revised SILS measure. Item reduction procedures along with reliability and validity data are reported in the Results. In addition, two versions of the adapted SILS were created, which included different referents. In one version, items referenced EBP generally (e.g., “Our principal is knowledgeable about evidence-based practice”). In the other, items referenced the specific EBP being implemented (e.g., “Our principal is knowledgeable about SW-PBIS”). Multigroup models were examined to determine whether the underlying factor structure was invariant across these two referents (see “Results” section).

#### Multifactor Leadership Questionnaire (MLQ)

The MLQ, a widely used measure of organizational leadership [20], was included to assess SILS convergent validity. Only the transformational and transactional leadership subscales were used in the present study. Transformational leadership is measured via five subscales: intellectual stimulation, inspirational motivation, individualized consideration, idealized behaviors, and idealized attributes. Two subscales comprise transactional leadership (contingent reward, management-by-exception active). The MLQ and its subscales have previously demonstrated strong psychometric properties [72, 73]. Internal consistency for subscales and scale scores in the current study were acceptable and as follows: intellectual stimulation (α = .88), inspirational motivation (α = .89), individualized consideration (α = .80), idealized behaviors (α = .84), idealized attributes (α = .84), transformational leadership total score (α = .91), contingent rewards (α = .78), management-by-exception active (α = .79).

#### Public School Teacher Questionnaire

The Public School Teacher Questionnaire (PSTQ), included for decades as part of the Schools and Staff Survey conducted by the National Center on Educational Statistics [74], was prioritized in the present study for purposes of divergent validity as a measure of teachers’ general attitudes toward teaching. Respondents used a 4-point Likert scale ranging from strongly disagree to strongly agree to rate 9 items that assess different attitudes toward the teaching profession (e.g., “The teaching profession is something that I enjoy and feel competent doing”). The PTSQ has demonstrated acceptable psychometric properties in extant research [75], as well as in the present study (α = .81).

### Data analytic approach

Several methodological approaches were employed to establish construct validity. Although this study did not have sufficient higher-level units (i.e., schools) to examine a multi-level confirmatory factor analysis (CFA), ICCs for SILS subscales provide evidence that 30–45% of the variability existed between schools, which is the level at which the construct theoretically resides. A series of CFAs were examined in M*plus* v8 [76] specifying robust standard errors to account for clustering of teachers within schools and weighted least squares means and variances (WLSMV) estimation with delta parameterization for the order-categorical scale items. Model fit was assessed using several indices including chi-square test statistics, comparative fit index (CFI) [77], the Tucker-Lewis index (TLI) [78], the root mean square error of approximation (RMSEA) [79, 80], and the standardized root mean square residual (SRMR) [77]. CFI and TLI values greater than .95, RMSEA less than or equal to .05, and SRMR less that or equal to .08 indicate a model well fit to the data. Standardized factor loadings (*β*) less than .55 were considered low and flagged for further examination [81].

Two measurement models were examined. The first included only first-order factors modeling exogenous, but correlated SILS subscales. The second model tested a second-order factor structure in which all first-order factors were then assumed to load onto the higher-order Implementation Leadership factor. Each of these models were tested twice—once prior to and once post item reduction (see description below). If the first-order factors appreciably load onto the higher-order factor, the second-order factor structure would be prioritized in alignment with this study’s driving theory, measurement development process, and goal of producing a brief yet comprehensive measure of a school’s strategic implementation leadership supportive of EBP implementation.

The initial CFAs were intended to provide evidence of the underlying measurement structure of the SILS. Once established, item characteristics curves were evaluated to narrow SILS items to those most representative of each subscale [82]. Item coverage and redundancy of information were assessed to reduce the number of items for each subscale to three, as the fewest items necessary is a recommended criterion for pragmatic measures [29]. Note that one subscale (proactive) included only three items and so was not subjected to item reduction. Using the reduced item version of SILS, we then tested both CFA models again and recalculated internal consistency estimates. Next, multigroup modeling was used to determine whether the underlying factor structure of SILS was invariant across versions of the scale employing general versus specific EBP item referents. Because the chi-square difference test is heavily influenced by sample size [83], two additional statistics were used to examine invariance across survey type. Cochran’s Q statistic [84] was used to determine the difference in magnitude between factor loadings of the two survey types, whereas *d* (Cox) was used to assess the difference in magnitude between thresholds. *Q* statistics that cluster around 0 indicate no substantive difference between factor loadings. There are not agreed upon cutpoints for *d*(Cox). Because *d*(Cox) ranges from 0 to 1, we employed a decision rule in line with similar effect sizes [85] such that values greater than .50 would be flagged as a moderate difference between thresholds of the two survey types that would require more thorough investigation.

Convergent and divergent validity were assessed via correlations between SILS subscales and select measures that were theoretically hypothesized to yield small-to-moderate (convergent) or no (divergent) association. Specifically, correlations between SILS subscales and correlations between SILS and MLQ subscales were examined to establish convergent validity. The SILS subscales theoretically measure a unitary construct and as such the inter-subscale correlations were anticipated to be moderate-to-large. Correlations between SILS and all MLQ subscales except for management-by-exception were also expected to be moderate-to-large, but smaller than the SILS inter-subscale correlations. Management-by-exception was anticipated to either be minimally or uncorrelated with SILS subscales. Divergent validity was similarly assessed via correlations, but between SILS subscales and both the PSTQ total score and school-level demographic characteristics. While the SILS and PTSQ are intended to measure different traits, they share the same assessment method (teacher reports) which makes it likely the two measures would share low-to-moderate correlations [86]. Some school-level demographic characteristics might influence teachers’ views of, experience with, and implementation of EBPs. As such, we hypothesized null-to-low correlations between SILS subscales and school-level demographics.

## Results

### Preliminary confirmatory factor analyses

To establish evidence of the hypothesized measurement model, two preliminary CFA models were examined using all 30 items of the adapted SILS. Results indicated acceptable and identical fit for both the seven-factor correlated model and the second-order factor in which the seven first-order factors loaded onto one higher order factor (CFI = .98, TLI = .98, RMSEA = .07, SRMR = .03). First-order factor loadings were appreciable (λ = .88 to λ = .99) and inter-factor correlations were high (*r* = .82 to .95). Second-order factor loadings were also high, ranging from λ = .92 to λ = .97.

### Item reduction

**Fig. 1:**
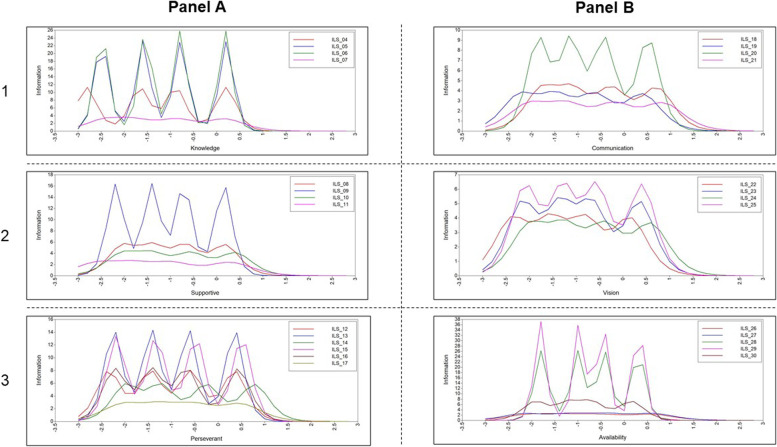
Item characteristics curves for all items by SILS subscale

Figure 1 presents item characteristics curves for all items by SILS subscale. The seventh item on the knowledgeable subscale (panel A, row 1) provided substantially less information (fewer and less pronounced peaks) than the other three items and had the lowest factor loading within the subscale. Item 11 on the supportive subscale (panel A, row 2) contributed less information than items 8, 9, and 10. However, item 11 is the only Supportive subscale item about direct use of an EBP—a key aspect of overall support of EBP implementation. Items 8 and 10 both elicit feedback regarding learning about an EBP, which duplicates content coverage. Further, item 10 showed a similar pattern while contributing less overall information than item 8. Items 12 and 16 on the perseverant subscale (panel A, row 3) provided a similar pattern, but less information than items 13 and 15. Item 17 provided little information and had the lowest factor loading within the subscale. For these reasons, items 7, 10, 12, 16, and 17 were all dropped from their respective subscales.

Item 21 on the communication subscale (panel B, row 1) provided less information and had a lower factor loading than the other three items in the subscale. Item 22 on the vision subscale (panel B, row 2) provided similar patterns of information to item 24, though had the lowest factor loading in the subscale. Items 26 and 27 on the availability subscale (panel B, row 3) provided substantially less information than the other items in the subscale as evidenced by relatively flat lines compared to the other items. For these reasons, items 21, 22, 26, and 27 were dropped from their respective subscales. Table 2 displays summary statistics and inter-item reliabilities for each SILS subscale and Table 3 displays individual item response frequencies.

**Table 2:** Summary statistics for School Implementation Leadership (SILS) subscales

ILS subscale	*n*, *x* ± sd	Cronbach’s α
Proactive	441, 2.82 ± 0.90	.92
Knowledgeable	441, 3.10 ± 0.88	.96
Supportive	441, 3.09 ± 0.92	.91
Perseverant	441, 2.85 ± 0.95	.94
Communication	441, 2.81 ± 0.99	.92
Vision/mission	441, 2.89 ± 0.98	.92
Availability	441, 2.93 ± 1.09	.96

**Table 3:** Response frequencies for School Implementation Leadership Scale (SILS) items

SILS subscale	Not at all *n*, %	Slight extent *n*,%	Moderate extent *n*,%	Great extent *n*,%	Very great extent *n*,%
Proactive					
Our principal has developed a plan to facilitate implementation of EBP.	6, 1.4	32, 7.3	82, 18.6	199, 45.1	122, 27.7
Our principal has removed obstacles to the implementation of EBP.	12, 2.7	33, 7.5	126, 28.6	176, 39.9	94, 21.3
Our principal has established clear school standards and expectations for the implementation of EBP.	11, 2.5	28, 6.3	101, 22.9	169, 38.3	132, 29.9
Knowledgeable					
Our principal is knowledgeable about EBP.	3, 0.7	23, 5.2	59, 13.4	184, 41.7	172, 39.0
Our principal is able to answer questions about EBP.	6, 1.4	22, 5.0	69, 15.6	175, 39.7	169, 38.3
Our principal knows what he or she is talking about when it comes to EBP.	6, 1.4	24, 5.4	65, 14.7	173, 39.2	173, 39.2
Supportive					
Our principal recognizes and appreciates teacher/school staff efforts toward successful implementation of EBP.	15, 3.4	29, 6.6	67, 15.2	142, 32.2	188, 42.6
Our principal supports teacher/school staff efforts to learn more about EBP.	9, 2.0	30, 6.8	62, 14.1	149, 33.8	191, 43.3
Our principal supports teacher/school staff efforts to use EBP.	7, 1.6	19, 4.3	59, 13.4	180, 40.8	176, 39.9
Perseverant					
Our principal carries on through the challenges of implementing EBP.	10, 2.3	28, 6.3	68, 15.4	181, 41.0	154, 34.9
Our principal effectively addresses critical issues regarding the implementation of EBP.	18, 4.1	34, 7.7	118, 26.8	176, 39.9	95, 21.5
Our principal consistently supports EBP implementation when confronted with setbacks.	11, 2.5	32, 7.3	85, 19.3	185, 42.0	128, 29.0
Communication					
Our principal establishes clear communication systems about EBP.	26, 5.9	38, 8.6	106, 24.0	162, 36.7	109, 24.7
Our principal talks about EBP.	11, 2.5	27, 6.1	76, 17.2	170, 38.5	157, 35.6
Our principal encourages others to communicate with her/him about EBP implementation.	20, 4.5	38, 8.6	84, 19.0	167, 37.9	132, 29.9
Vision/mission					
Our principal links the implementation of EBP to improved student outcomes.	13, 2.9	34, 7.7	65, 14.7	168, 38.1	161, 36.5
Our principal has a clear vision for the implementation of EBP in this school.	19, 4.3	36, 8.2	100, 22.7	161, 36.5	125, 28.3
Our principal connects EBP to the broader mission of our school.	14, 3.2	34, 7.7	77, 17.5	166, 37.6	150, 34.0
Availability					
Our principal is accessible if I need help with implementing EBP.	20, 4.5	45, 10.2	75, 17.0	137, 31.1	164, 37.2
Our principal is available to discuss implementation of EBP.	19, 4.3	49, 11.1	68, 15.4	142, 32.2	163, 37.0
If I have a problem or concern regarding EBP, I can contact our principal.	17, 3.9	34, 7.7	48, 10.9	148, 33.6	194, 44.0

### Confirmatory factor analyses post-item reduction

**Fig. 2:**
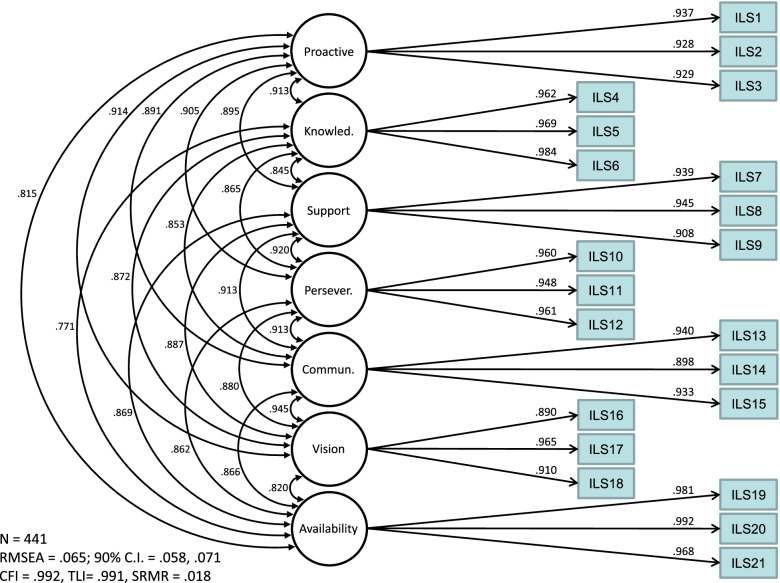
First-order SILS factor loadings

**Fig. 3:**
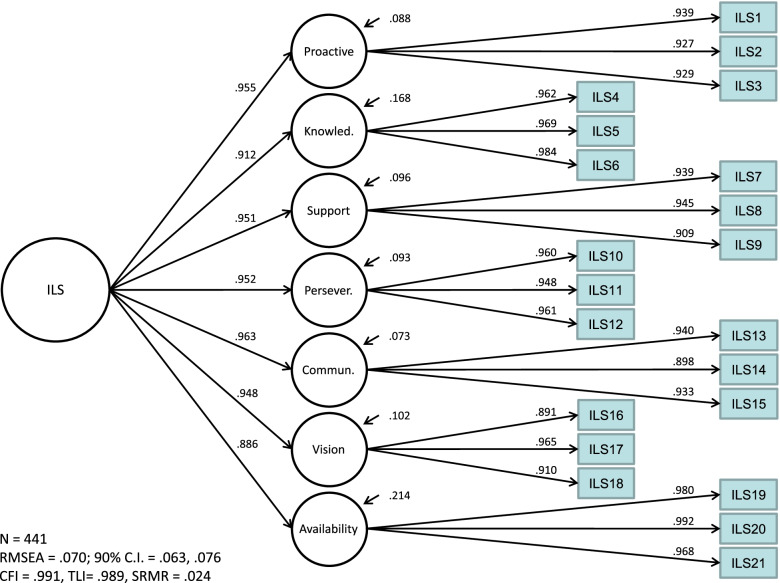
Second-order SILS factor loadings

To confirm that the measurement structure observed using all 30 adapted SILS items held post-item reduction, first- and second-order CFAs were examined using the reduced 21-item scale (3 items per subscale). Results again indicated acceptable and identical fit for both the seven-factor correlated and the second-order factor models (CFI = .99, TLI = .99, RMSEA = .07, SRMR = .02). Figure 2 shows that first-order factor loadings were appreciable (λ = .89 to λ = .98), and inter-factor correlations were high (*r* = .77 to .95). Second-order factor loadings were also high, ranging from λ=.89 to λ=.96 (see Fig. 3), providing evidence supportive of the theoretical model that SILS subscales comprise a higher-order Implementation Leadership factor. School-specific means, medians, modes, standard deviations, and ranges for each final SILS subscale are provided in Additional file 1.

### Multigroup model to test invariance

Results from multigroup models testing invariance between all paths freely estimated versus all paths constrained to be equal indicated invariance across the two survey types administered (*χ*2(105) = 127.33, *p* ≤ .07). The chi-square statistic is sensitive to sample size so invariance was also examined using *q* and d(Cox) statistics, which probe where invariance might be located (factor loadings, thresholds) and the magnitude of the invariance. Results supported invariance of factor loadings as evidenced by *q* statistics clustered around zero (*q* = − .03–.02). The vast majority of d(Cox) statistics revealed no difference in thresholds between the two survey types, and no values greater than .50 were observed. There were five thresholds (items 9, 23, 29, and two thresholds for item 30) that approached (.40–.48) though did not reach the .50 cutpoint. The pattern of results indicated that respondents to the general version of the survey may have been more likely to endorse items 29 and 30 than respondents to the specific version. Though future refinement and replication may be needed (see “Discussion” section), the preponderance of evidence showed SILS factor loadings (i.e., the amount of variance each item contributes to a latent factor) and thresholds (i.e., the intercept for each categorical response option) to be invariant across survey type.

### Convergent and divergent validity

Table 4 displays bivariate correlations between the means for each of the three-item SILS subscales and other measures included to evidence convergent or divergent validity. Measures of convergent validity are included closer to the top of the table so correlations should decrease as one scans down. As hypothesized and aligned with the inter-factor correlations observed in CFA results, the bivariate correlations between SILS subscales were high (*r* = .71–.93). Also aligned with hypotheses, all MLQ subscale and scale scores except for management-by-exception were moderately to highly correlated with SILS subscales (*r* = .56–.80), and less so than the SILS inter-subscale correlations. In line with expectations, Management-by-Exception shared low to no correlation with SILS subscales (*r* = .05–.12). Correlations with the PTSQ provide preliminary evidence of divergent validity, with moderate correlations that are lower than most subscales of the MLQ (*r* = .36 – .44). Finally, and providing additional evidence of divergent validity, correlations between SILS subscales and school demographics were low (*r* = − .31 – .22).

**Table 4:** Correlations among theoretically related and unrelated variables

		**SILS**^a^							
**Convergent**	**SILS**^a^	A	B	C	D	E	F	G	H
	A. Proactive	1.00							
	B. Knowledgeable	.834^e^	1.00						
	C. Supportive	.815^e^	.781^e^	1.00					
	D. Perseverant	.841^e^	.801^e^	.858^e^	1.00				
	E. Communication	.816^e^	.782^e^	.844^e^	.846^e^	1.00			
	F. Vision/mission	.830^e^	.794^e^	.819^e^	.817^e^	.868^e^	1.00		
	G. Availability	.755^e^	.714^e^	.817^e^	.816^e^	.809^e^	.760^e^	1.00	
	H. Total	.917^e^	.886^e^	.926^e^	.933^e^	.933^e^	.919^e^	.891^e^	1.00
	**MLQ**^b^								
	Intellectual stimulation	.652^e^	.632^e^	.691^e^	.687^e^	.686^e^	.679^e^	.696^e^	.737^e^
	Inspirational motivation	.633^e^	.643^e^	.700^e^	.657^e^	.642^e^	.686^e^	.630^e^	.717^e^
	Individualized consideration	.632^e^	.618^e^	.690^e^	.660^e^	.650^e^	.640^e^	.704^e^	.719^e^
	Idealized Influence (behavior)	.654^e^	.638^e^	.697^e^	.673^e^	.650^e^	.687^e^	.622^e^	.721^e^
	Idealized Influence (attributed)	.654^e^	.673^e^	.726^e^	.696^e^	.681^e^	.672^e^	.717^e^	.753^e^
	Transformation leadership total	.704^e^	.698^e^	.766^e^	.737^e^	.726^e^	.736^e^	.739^e^	.798^e^
	Management-by-exception (active)	.116^d^	.108^d^	.049	.059	.100^d^	.116^d^	.070	.096^e^
	Contingent reward	.586^e^	.563^e^	.627^e^	.606^e^	.625^e^	.640^e^	.652^e^	.673^e^
**Divergent**	**PSTQ**^c^								
	Total	.378^e^	.362^e^	.440^e^	.400^e^	.397^e^	.391^e^	.412^e^	.435^e^
	**School demographics**								
	School size	.111^d^	.091	.119^d^	.134^e^	.118^d^	.136^e^	.046	.117^d^
	% White	.166^e^	.171^e^	.245^e^	.217^e^	.177^e^	.164^e^	.213^e^	.212^e^
	% Non-White	– .241^e^	– .254^e^	– .302^e^	– .311^e^	– .258^e^	– .234^e^	– .243^e^	– .288^e^
	% Transitional bilingual	.058	.040	– .005	– .001	– .027	– .005	– .052	– .001
	% Special education	– .072	– .088	– .088	– .082	– .081	– .122^d^	– .098^d^	– .099^d^
	% Attendance rates	.024	.047	.017	.051	.078	.072	.032	.050

^a^ = School Implementation Leadership Scale; ^b^ = Multifactor Leadership Questionnaire; ^c^ = Public School Teacher Questionnaire

^d^Correlation is significant at the 0.05 level (2-tailed)

^e^Correlation is significant at the .01 level (2-tailed)

## Discussion

The objectives of the current study were to develop and test a revised version of the SILS for use in the education sector by completing item reduction, confirmatory factor analyses, and examinations of convergent and divergent validity in the context of universal, evidence-based mental health prevention program implementation. Results provided strong evidence of structural validity for the 21-item, 7-factor SILS following item reduction, including for three newly developed subscales that represent salient implementation leadership characteristics in schools: communication, vision, and availability. Communication involves concrete efforts to engage in bidirectional communication surrounding EBP implementation and often is a foundation on which other implementation leadership behaviors are built and maintained. Vision reflects how a leader integrates EBP implementation with the core objectives of a school. Finally, Availability is the extent to which leaders are accessible and responsive to staff needs or problems surrounding implementation. Convergent and divergent validity results for all SILS subscales confirmed study hypotheses, including higher correlations with general leadership scales relative to teacher attitudes and school demographics. Measuring these aspects of implementation leadership provides additional avenues for tracking and supporting the behaviors of school building-level leaders interested in improving the availability of EBP in their systems.

Scores on the SILS suggested moderate levels of most implementation leadership constructs in the current sample (Table 2). All values on the original subscales (i.e., proactive, knowledgeable, supportive, perseverant) were consistently higher than the original ILS development sample in outpatient mental health [26] and validations in substance use treatment settings [31] and acute care [32], but generally comparable to a validation study in child welfare [30]. Scores also were higher than those observed on the earlier version of the ILS adapted for schools [66]. This could be due to the fact that the items and subscales in the new SILS had been specifically tailored to reflect the implementation experiences of school personnel, potentially improving their likelihood of endorsement.

We also observed evidence of invariance in the factor structures between the general (“evidence-based practice” referent) and intervention-specific (SWPBIS or PATHS referent) versions of the SILS. This suggests that the instrument can likely be used to assess the implementation supports delivered by leaders for individual interventions as well as across multiple EBPs, though future replication and refinement are prudent given the moderate to large effect sizes observed for five thresholds (out of 120). Importantly, the invariance established for the SILS factor loadings provides compelling evidence that the interpretation of the underlying construct is synonymous across the different referents used. Differences in thresholds, which were minimal, indicate a shift in the response curves to the right or left of a distribution, but have no bearing on the interpretation of the underlying construct. Such invariance may support the use of the general version of the SILS prior to the selection of an EBP to implement, such as during the Exploration phase of implementation [10], and the intervention-specific version in later phases. However, future research is needed to evaluate whether the predictive validity of the SILS for variables such as implementation climate [87] and implementation outcomes [88] is equivalent for the general and specific versions.

### Implications for supporting implementation leadership in schools

Leadership has been found to be a significant predictor of organizational climate [25]. In schools, aspects of leadership and climate also are associated with student wellbeing and success [58, 89]. Measuring implementation leadership in the education sector can be useful in supporting leadership behaviors that create a conducive implementation climate across phases of implementation, including prior to or during active EBP implementation and sustainment. However, little research has developed and evaluated specific implementation strategies that focus on changing aspects of the school organizational context to cultivate an environment that influences educators’ adoption, use, and sustainment of EBPs. Principals and educational leadership teams typically receive little explicit guidance or support surrounding EBP implementation. To address this, the SILS could form the foundation of a leadership-oriented action planning process in schools to improve organizational readiness (i.e., an organization’s commitment to change and implement new practices) [90]. Action planning involves determining who is going to do what and along what sequence and timeline in order for an organization to advance its strategic goals [91]. Low initial readiness accounts for over half of all unsuccessful organizational change efforts [92] and is heavily influenced by leadership. Since the SILS is pragmatic, brief, and has been designed for repeated administration, resulting data could be used in the context of action planning to drive deployment of novel implementation strategies based on context-specific needs identified prior to, or over the course of, implementation. Future studies should investigate the relative utility of different methods of data presentation—and different indicators of central tendency (e.g., mean vs. median)—for feedback and action planning processes.

Existing leadership-focused implementation strategies, such as Leadership and Organizational Change for Implementation (LOCI) [28] or iLead [93] also likely have utility for promoting implementation leadership behaviors among principals and other school building-level leaders. For instance, our research team is currently leveraging the SILS in an adaptation of LOCI for building-level leaders who are implementing mental health prevention programs in schools (Institute of Education Sciences award number R305A200023; https://ies.ed.gov/funding/grantsearch/details.asp?ID=4471). Components of the strategy are being modified to fit with contextual factors such as the school academic calendar, existing professional development needs and supports for leaders, and policies surrounding the design and execution of school improvement plans.

## Limitations and future directions

The current study provides strong evidence for the structural, convergent, and divergent validity of the SILS among a sample of elementary school teachers delivering universal prevention programming focused on children’s mental health. Nonetheless, there are important limitations and opportunities for future research surrounding the evaluation of implementation leadership in schools. First, although data were collected during the implementation of two different prevention programs, a larger number of participants were implementing SWPBIS. Additional studies should continue to expand the application of the general and specific versions of the SILS to other programs. Second, as noted above, additional research is needed to evaluate the predictive validity of the SILS, as it relates to variables such as implementation climate and implementation outcomes (e.g., EBP fidelity). Third, the current study focused on principals as the primary formal leaders in school buildings who are ideal targets to promote strategic implementation leadership, given their accountability and central role in decision-making. However, other informal leaders often play important roles in the implementation of EBPs in educational settings. Future studies with the SILS might incorporate additional informal, building-level leaders into data collection efforts in schools that support distributed leadership models [94]. Fourth, further research should examine the degree to which the additional implementation leadership dimensions in the SILS (i.e., communication, vision, and availability) generalize to other settings such as medical care, behavioral health, addiction, or child welfare. Fifth, the number of respondents per organizational unit used to assess organizational constructs such as leadership and climate has been found to vary in the implementation literature [95]. To promote efficient evaluation and feedback processes, future research with the SILS and could explicitly assess the minimum number of responses needed to produce a reliable and valid assessment. Finally, the current study conceptualized implementation leadership as an organizational construct, similar to prevailing characterizations of other constructs such as organizational climate. Nevertheless, although we evaluated ICCs to examine between-school variability, the study was not sufficiently powered to rigorously examine the measurement model at the school level.

## Conclusion

The current study adapted and expanded a leading instrument for measuring strategic implementation leadership to ensure its relevance to the implementation of universal prevention programs in schools. The resulting SILS demonstrated structural, convergent, and divergent validity in the context of two distinct interventions designed to prevent student mental health problems. As the education sector continues to be the most common location in the USA for the delivery of mental health services to children and adolescents [4], opportunities to understand and support building-level leaders in promoting the use of EBP in their systems is critical to ensuring public health impact.

## Supplementary Information

**Additional file 1.**

## Data Availability

The datasets generated and/or analyzed during the current study are not publicly available but are available from the corresponding author on reasonable request.

## Abbreviations

CFA: Confirmatory factor analysis
CFI: Comparative fit index
EBP: Evidence-based practice
ILS: Implementation Leadership Scale
LOCI: Leadership and Organizational Change for Implementation
MLQ: Multifactor Leadership Questionnaire
MTSS: Multi-tiered systems of support
PATHS: Promoting Alternative Thinking Strategies
PSTQ: Public School Teacher Questionnaire
RMSEA: Root mean square error of approximation
SILS: School Implementation Leadership Scale
SRMR: Standardized root mean square residual
SWPBIS: Schoolwide Positive Behavioral Interventions and Supports
TLI: Tucker-Lewis Index
WLSMV: Weighted least squares means and variances
