# Supplementary material for: Leading the charge in the education sector: development and validation of the School Implementation Leadership Scale (SILS)
Source: Implement Sci. 2022 Jul 19;17:48. doi: 10.1186/s13012-022-01222-7 (PMC9295535; doi:10.1186/s13012-022-01222-7)
Supplement: Supplementary file 1 — Additional file 1. [file 13012_2022_1222_MOESM1_ESM.docx]

Additional File 1

Univariate statistics for School Implementation Leadership subscales and total score by school.

| School | *N* Teachers | Variables | Mean | Median | Mode | Std. Dev. | Minimum | Maximum |
| --- | --- | --- | --- | --- | --- | --- | --- | --- |
| 1 | 9 | Availability | 2.70 | 3.00 | 3.00 | 0.92 | 0.67 | 4.00 |
|  |  | Communication | 2.41 | 2.33 | 2.00 | 0.70 | 1.33 | 3.67 |
|  |  | Knowledge | 3.19 | 3.00 | 3.00 | 0.53 | 2.33 | 4.00 |
|  |  | Perseverance | 2.59 | 2.67 | 3.00 | 0.68 | 1.33 | 3.67 |
|  |  | Proactive | 2.67 | 2.33 | 2.00 | 0.91 | 1.33 | 4.00 |
|  |  | Support | 2.63 | 2.33 | 1.67 | 0.86 | 1.67 | 4.00 |
|  |  | Vision | 2.48 | 2.33 | 2.00 | 0.69 | 1.33 | 3.67 |
|  |  | ILS (Total) | 2.67 | 2.57 | . | 0.68 | 1.57 | 3.86 |
| 2 | 9 | Availability | 2.70 | 3.00 | 3.00 | 0.81 | 1.00 | 4.00 |
|  |  | Communication | 2.70 | 2.67 | 2.67 | 0.77 | 1.33 | 4.00 |
|  |  | Knowledge | 3.26 | 3.33 | 3.00 | 0.57 | 2.33 | 4.00 |
|  |  | Perseverance | 2.81 | 2.67 | 2.00 | 0.78 | 2.00 | 4.00 |
|  |  | Proactive | 2.89 | 3.00 | 2.33 | 0.85 | 1.33 | 4.00 |
|  |  | Support | 3.04 | 3.00 | 2.33 | 0.63 | 2.33 | 4.00 |
|  |  | Vision | 2.81 | 3.00 | 3.00 | 0.85 | 1.00 | 4.00 |
|  |  | ILS (Total) | 2.89 | 3.00 | 3.29 | 0.67 | 1.62 | 3.95 |
| 3 | 10 | Availability | 1.57 | 1.83 | 0.00 | 1.26 | 0.00 | 3.33 |
|  |  | Communication | 1.80 | 2.00 | 2.33 | 1.04 | 0.00 | 3.67 |
|  |  | Knowledge | 2.33 | 2.67 | 3.00 | 0.82 | 1.00 | 3.00 |
|  |  | Perseverance | 1.93 | 2.00 | 2.00 | 0.89 | 0.67 | 3.00 |
|  |  | Proactive | 2.13 | 2.17 | 1.00 | 0.83 | 1.00 | 3.33 |
|  |  | Support | 1.87 | 2.00 | 2.00 | 0.97 | 0.33 | 3.33 |
|  |  | Vision | 2.13 | 2.67 | 2.67 | 1.23 | 0.00 | 3.67 |
|  |  | ILS (Total) | 1.97 | 2.38 | 0.67 | 0.92 | 0.67 | 3.05 |
| 4 | 9 | Availability | 3.26 | 4.00 | 4.00 | 1.51 | 0.00 | 4.00 |
|  |  | Communication | 3.19 | 4.00 | 4.00 | 1.11 | 1.00 | 4.00 |
|  |  | Knowledge | 3.44 | 4.00 | 4.00 | 0.73 | 2.33 | 4.00 |
|  |  | Perseverance | 3.11 | 3.67 | 4.00 | 1.26 | 0.33 | 4.00 |
|  |  | Proactive | 3.11 | 3.33 | 4.00 | 0.99 | 1.67 | 4.00 |
|  |  | Support | 3.56 | 4.00 | 4.00 | 0.80 | 1.67 | 4.00 |
|  |  | Vision | 3.37 | 3.67 | 4.00 | 0.89 | 1.33 | 4.00 |
|  |  | ILS (Total) | 3.29 | 3.90 | 3.90 | 0.92 | 1.43 | 4.00 |
| 5 | 7 | Availability | 3.14 | 3.33 | 4.00 | 0.98 | 1.67 | 4.00 |
|  |  | Communication | 3.29 | 3.33 | 3.33 | 0.73 | 2.00 | 4.00 |
|  |  | Knowledge | 3.43 | 3.67 | 3.00 | 0.53 | 2.67 | 4.00 |
|  |  | Perseverance | 3.05 | 3.00 | 3.00 | 0.62 | 2.00 | 4.00 |
|  |  | Proactive | 3.10 | 3.00 | 3.00 | 0.53 | 2.33 | 4.00 |
|  |  | Support | 3.05 | 3.33 | 4.00 | 0.95 | 1.67 | 4.00 |
|  |  | Vision | 3.24 | 3.67 | 3.67 | 0.98 | 1.67 | 4.00 |
|  |  | ILS (Total) | 3.18 | 3.43 | 3.43 | 0.68 | 2.14 | 3.86 |
| 6 | 8 | Availability | 2.38 | 2.67 | 1.00 | 1.55 | 0.00 | 4.00 |
|  |  | Communication | 2.58 | 3.00 | 3.00 | 1.23 | 0.67 | 4.00 |
|  |  | Knowledge | 2.92 | 3.00 | 3.00 | 1.00 | 1.00 | 4.00 |
|  |  | Perseverance | 2.71 | 3.17 | 2.00 | 1.34 | 0.00 | 4.00 |
|  |  | Proactive | 2.92 | 3.00 | 3.00 | 0.92 | 1.33 | 4.00 |
|  |  | Support | 2.88 | 3.17 | 4.00 | 1.15 | 1.00 | 4.00 |
|  |  | Vision | 2.33 | 2.33 | 2.33 | 1.43 | 0.00 | 4.00 |
|  |  | ILS (Total) | 2.67 | 3.00 | . | 1.18 | 0.67 | 4.00 |
| 7 | 8 | Availability | 3.71 | 4.00 | 4.00 | 0.45 | 3.00 | 4.00 |
|  |  | Communication | 3.42 | 3.50 | 4.00 | 0.61 | 2.33 | 4.00 |
|  |  | Knowledge | 3.67 | 4.00 | 4.00 | 0.53 | 2.67 | 4.00 |
|  |  | Perseverance | 3.63 | 3.83 | 4.00 | 0.45 | 3.00 | 4.00 |
|  |  | Proactive | 3.58 | 3.67 | 4.00 | 0.46 | 2.67 | 4.00 |
|  |  | Support | 3.79 | 4.00 | 4.00 | 0.35 | 3.00 | 4.00 |
|  |  | Vision | 3.46 | 3.67 | 4.00 | 0.67 | 2.33 | 4.00 |
|  |  | ILS (Total) | 3.61 | 3.62 | 3.19 | 0.36 | 3.19 | 4.00 |
| 8 | 11 | Availability | 3.42 | 3.00 | 3.00 | 0.56 | 2.67 | 4.00 |
|  |  | Communication | 3.36 | 3.33 | 3.00 | 0.57 | 2.33 | 4.00 |
|  |  | Knowledge | 3.67 | 4.00 | 4.00 | 0.45 | 3.00 | 4.00 |
|  |  | Perseverance | 3.27 | 3.33 | 3.00 | 0.61 | 2.00 | 4.00 |
|  |  | Proactive | 3.42 | 3.33 | 3.33 | 0.52 | 2.33 | 4.00 |
|  |  | Support | 3.73 | 3.67 | 3.67 | 0.29 | 3.00 | 4.00 |
|  |  | Vision | 3.64 | 4.00 | 4.00 | 0.48 | 2.67 | 4.00 |
|  |  | ILS (Total) | 3.50 | 3.62 | 3.62 | 0.43 | 2.86 | 4.00 |
| 9 | 10 | Availability | 2.93 | 3.17 | 2.00 | 0.98 | 1.00 | 4.00 |
|  |  | Communication | 2.40 | 2.33 | 1.33 | 1.05 | 1.00 | 4.00 |
|  |  | Knowledge | 2.60 | 2.67 | 2.00 | 0.64 | 2.00 | 4.00 |
|  |  | Perseverance | 2.60 | 2.50 | 2.00 | 0.80 | 1.33 | 4.00 |
|  |  | Proactive | 2.67 | 2.67 | 2.00 | 0.79 | 1.33 | 4.00 |
|  |  | Support | 2.87 | 2.83 | 4.00 | 1.04 | 1.00 | 4.00 |
|  |  | Vision | 2.40 | 2.33 | 2.33 | 0.77 | 1.33 | 4.00 |
|  |  | ILS (Total) | 2.64 | 2.45 | . | 0.73 | 1.33 | 4.00 |
| 10 | 7 | Availability | 2.90 | 3.00 | 3.00 | 1.07 | 1.00 | 4.00 |
|  |  | Communication | 2.71 | 3.00 | 3.00 | 0.87 | 1.00 | 3.67 |
|  |  | Knowledge | 3.00 | 3.00 | 4.00 | 1.15 | 1.00 | 4.00 |
|  |  | Perseverance | 2.38 | 2.67 | 2.67 | 0.99 | 1.00 | 3.67 |
|  |  | Proactive | 2.52 | 2.67 | 2.00 | 0.69 | 1.67 | 3.67 |
|  |  | Support | 3.10 | 3.33 | 3.00 | 0.85 | 1.33 | 4.00 |
|  |  | Vision | 2.71 | 2.67 | 2.67 | 0.99 | 1.00 | 4.00 |
|  |  | ILS (Total) | 2.76 | 2.81 | . | 0.85 | 1.19 | 3.71 |
| 11 | 9 | Availability | 3.67 | 4.00 | 4.00 | 0.44 | 3.00 | 4.00 |
|  |  | Communication | 3.00 | 3.00 | 3.00 | 0.78 | 2.00 | 4.00 |
|  |  | Knowledge | 3.48 | 3.33 | 3.00 | 0.50 | 3.00 | 4.00 |
|  |  | Perseverance | 3.26 | 3.00 | 3.00 | 0.43 | 3.00 | 4.00 |
|  |  | Proactive | 3.26 | 3.00 | 3.00 | 0.64 | 2.00 | 4.00 |
|  |  | Support | 3.37 | 3.00 | 3.00 | 0.54 | 2.67 | 4.00 |
|  |  | Vision | 3.30 | 3.00 | 4.00 | 0.73 | 2.00 | 4.00 |
|  |  | ILS (Total) | 3.33 | 3.14 | . | 0.53 | 2.57 | 4.00 |
| 12 | 9 | Availability | 1.48 | 1.67 | 1.67 | 1.19 | 0.00 | 4.00 |
|  |  | Communication | 1.33 | 1.00 | 2.33 | 1.09 | 0.00 | 2.67 |
|  |  | Knowledge | 1.85 | 2.00 | 2.00 | 0.80 | 0.67 | 3.00 |
|  |  | Perseverance | 1.56 | 1.33 | 0.67 | 1.09 | 0.00 | 3.33 |
|  |  | Proactive | 1.70 | 2.00 | 2.00 | 0.59 | 1.00 | 2.67 |
|  |  | Support | 1.78 | 1.33 | 1.00 | 1.03 | 0.33 | 3.33 |
|  |  | Vision | 1.59 | 2.00 | 3.00 | 1.27 | 0.00 | 3.00 |
|  |  | ILS (Total) | 1.61 | 1.48 | . | 0.93 | 0.43 | 3.10 |
| 13 | 7 | Availability | 3.10 | 3.00 | 4.00 | 0.92 | 2.00 | 4.00 |
|  |  | Communication | 2.86 | 3.00 | 2.00 | 0.96 | 1.67 | 4.00 |
|  |  | Knowledge | 3.33 | 3.00 | 3.00 | 0.67 | 2.33 | 4.00 |
|  |  | Perseverance | 3.10 | 3.00 | 2.67 | 0.74 | 2.00 | 4.00 |
|  |  | Proactive | 3.00 | 3.00 | 2.33 | 0.61 | 2.33 | 4.00 |
|  |  | Support | 3.33 | 3.67 | 3.67 | 0.61 | 2.33 | 4.00 |
|  |  | Vision | 3.19 | 3.33 | 2.67 | 0.77 | 2.00 | 4.00 |
|  |  | ILS (Total) | 3.13 | 2.90 | 3.71 | 0.66 | 2.48 | 4.00 |
| 14 | 6 | Availability | 2.28 | 2.17 | 2.00 | 0.49 | 1.67 | 3.00 |
|  |  | Communication | 2.33 | 2.17 | 1.67 | 0.70 | 1.67 | 3.33 |
|  |  | Knowledge | 2.61 | 2.83 | 3.00 | 0.49 | 2.00 | 3.00 |
|  |  | Perseverance | 2.50 | 2.50 | 2.33 | 0.51 | 1.67 | 3.00 |
|  |  | Proactive | 2.17 | 2.33 | 2.33 | 0.62 | 1.00 | 2.67 |
|  |  | Support | 2.67 | 2.67 | 2.67 | 0.47 | 2.00 | 3.33 |
|  |  | Vision | 2.00 | 1.83 | . | 0.87 | 1.00 | 3.33 |
|  |  | ILS (Total) | 2.37 | 2.43 | . | 0.38 | 1.81 | 2.86 |
| 15 | 7 | Availability | 2.71 | 3.00 | 3.00 | 0.83 | 1.00 | 3.67 |
|  |  | Communication | 2.71 | 3.00 | 3.00 | 1.13 | 0.33 | 4.00 |
|  |  | Knowledge | 3.24 | 3.00 | 3.00 | 0.71 | 2.00 | 4.00 |
|  |  | Perseverance | 2.57 | 3.00 | 3.00 | 0.92 | 1.00 | 3.67 |
|  |  | Proactive | 2.57 | 2.67 | 2.33 | 0.53 | 1.67 | 3.33 |
|  |  | Support | 2.90 | 3.00 | 3.00 | 0.79 | 1.33 | 3.67 |
|  |  | Vision | 2.29 | 2.67 | 2.67 | 0.85 | 0.67 | 3.00 |
|  |  | ILS (Total) | 2.71 | 2.86 | . | 0.74 | 1.14 | 3.38 |
| 16 | 11 | Availability | 3.03 | 3.00 | 4.00 | 1.05 | 0.67 | 4.00 |
|  |  | Communication | 2.85 | 3.00 | 2.67 | 0.74 | 1.00 | 4.00 |
|  |  | Knowledge | 3.18 | 3.00 | 3.00 | 0.67 | 2.00 | 4.00 |
|  |  | Perseverance | 3.03 | 3.00 | 4.00 | 0.94 | 1.00 | 4.00 |
|  |  | Proactive | 3.00 | 3.00 | 3.00 | 0.83 | 1.00 | 4.00 |
|  |  | Support | 3.21 | 3.00 | 4.00 | 0.75 | 1.67 | 4.00 |
|  |  | Vision | 2.94 | 3.00 | 3.00 | 0.77 | 1.33 | 4.00 |
|  |  | ILS (Total) | 3.03 | 2.95 | 2.95 | 0.74 | 1.38 | 4.00 |
| 17 | 10 | Availability | 3.57 | 4.00 | 4.00 | 0.74 | 2.00 | 4.00 |
|  |  | Communication | 3.17 | 3.33 | 3.33 | 0.63 | 2.00 | 4.00 |
|  |  | Knowledge | 3.23 | 3.00 | 4.00 | 0.70 | 2.33 | 4.00 |
|  |  | Perseverance | 3.00 | 3.00 | 3.00 | 0.65 | 2.00 | 4.00 |
|  |  | Proactive | 3.03 | 3.17 | 3.00 | 0.81 | 1.67 | 4.00 |
|  |  | Support | 3.23 | 3.17 | 4.00 | 0.67 | 2.33 | 4.00 |
|  |  | Vision | 2.73 | 2.83 | 2.00 | 0.83 | 1.67 | 4.00 |
|  |  | ILS (Total) | 3.14 | 3.19 | 3.19 | 0.60 | 2.33 | 4.00 |
| 18 | 11 | Availability | 3.58 | 4.00 | 4.00 | 0.63 | 2.00 | 4.00 |
|  |  | Communication | 3.15 | 3.00 | 3.00 | 0.60 | 2.00 | 4.00 |
|  |  | Knowledge | 3.61 | 4.00 | 4.00 | 0.66 | 2.00 | 4.00 |
|  |  | Perseverance | 3.36 | 3.67 | 3.00 | 0.72 | 1.67 | 4.00 |
|  |  | Proactive | 3.30 | 3.33 | 4.00 | 0.71 | 2.00 | 4.00 |
|  |  | Support | 3.45 | 3.67 | 4.00 | 0.70 | 1.67 | 4.00 |
|  |  | Vision | 3.30 | 3.33 | 3.33 | 0.71 | 2.00 | 4.00 |
|  |  | ILS (Total) | 3.39 | 3.43 | 3.38 | 0.60 | 1.90 | 4.00 |
| 19 | 9 | Availability | 2.96 | 3.00 | 3.00 | 0.86 | 1.33 | 4.00 |
|  |  | Communication | 2.59 | 3.00 | 3.00 | 1.01 | 1.00 | 3.67 |
|  |  | Knowledge | 2.11 | 2.00 | 2.00 | 0.78 | 1.00 | 3.00 |
|  |  | Perseverance | 2.52 | 2.67 | 3.00 | 0.71 | 1.67 | 3.67 |
|  |  | Proactive | 2.19 | 2.33 | 3.00 | 0.78 | 1.00 | 3.00 |
|  |  | Support | 2.89 | 3.00 | 3.00 | 0.65 | 1.67 | 3.67 |
|  |  | Vision | 2.37 | 2.33 | 3.00 | 0.84 | 1.33 | 3.67 |
|  |  | ILS (Total) | 2.52 | 2.62 | . | 0.73 | 1.38 | 3.43 |
| 20 | 11 | Availability | 2.91 | 3.00 | 2.00 | 0.80 | 2.00 | 4.00 |
|  |  | Communication | 2.85 | 2.67 | 2.67 | 0.79 | 1.67 | 4.00 |
|  |  | Knowledge | 3.09 | 3.00 | 3.00 | 0.72 | 2.00 | 4.00 |
|  |  | Perseverance | 2.91 | 3.00 | 3.00 | 0.67 | 2.00 | 4.00 |
|  |  | Proactive | 2.85 | 2.67 | 2.00 | 0.82 | 2.00 | 4.00 |
|  |  | Support | 3.09 | 3.00 | 2.67 | 0.76 | 2.00 | 4.00 |
|  |  | Vision | 3.00 | 3.00 | 2.00 | 0.77 | 2.00 | 4.00 |
|  |  | ILS (Total) | 2.96 | 2.86 | . | 0.67 | 2.00 | 4.00 |
| 21 | 9 | Availability | 3.33 | 3.67 | 3.00 | 0.65 | 2.00 | 4.00 |
|  |  | Communication | 3.00 | 3.00 | 3.00 | 0.53 | 2.00 | 4.00 |
|  |  | Knowledge | 3.22 | 3.00 | 3.00 | 0.60 | 2.00 | 4.00 |
|  |  | Perseverance | 3.07 | 3.00 | 3.00 | 0.52 | 2.00 | 4.00 |
|  |  | Proactive | 2.89 | 3.00 | 3.00 | 0.65 | 2.00 | 4.00 |
|  |  | Support | 3.41 | 3.67 | 4.00 | 0.66 | 2.00 | 4.00 |
|  |  | Vision | 3.11 | 3.00 | 3.00 | 0.73 | 2.00 | 4.00 |
|  |  | ILS (Total) | 3.15 | 3.10 | 3.00 | 0.57 | 2.00 | 4.00 |
| 22 | 9 | Availability | 3.60 | 4.00 | 4.00 | 0.52 | 3.00 | 4.00 |
|  |  | Communication | 2.77 | 3.00 | 3.00 | 0.94 | 1.33 | 4.00 |
|  |  | Knowledge | 3.47 | 3.50 | 3.00 | 0.45 | 3.00 | 4.00 |
|  |  | Perseverance | 3.27 | 3.17 | 4.00 | 0.73 | 2.00 | 4.00 |
|  |  | Proactive | 2.93 | 3.00 | 3.00 | 0.58 | 2.00 | 4.00 |
|  |  | Support | 3.23 | 3.17 | 3.00 | 0.59 | 2.00 | 4.00 |
|  |  | Vision | 2.70 | 3.00 | 3.00 | 0.69 | 1.67 | 3.67 |
|  |  | ILS (Total) | 3.14 | 3.12 | . | 0.48 | 2.24 | 3.90 |
| 23 | 14 | Availability | 2.93 | 3.00 | 3.00 | 1.10 | 1.00 | 4.00 |
|  |  | Communication | 2.71 | 2.83 | 2.67 | 0.86 | 1.00 | 3.67 |
|  |  | Knowledge | 2.86 | 3.00 | 3.00 | 0.96 | 1.00 | 4.00 |
|  |  | Perseverance | 2.40 | 2.67 | 3.00 | 0.79 | 1.00 | 3.33 |
|  |  | Proactive | 2.62 | 3.00 | 3.00 | 0.87 | 1.00 | 4.00 |
|  |  | Support | 2.88 | 3.00 | 3.00 | 0.78 | 1.33 | 3.67 |
|  |  | Vision | 2.81 | 2.83 | 2.67 | 0.77 | 1.00 | 3.67 |
|  |  | ILS (Total) | 2.74 | 2.95 | 2.76 | 0.78 | 1.24 | 3.57 |
| 24 | 4 | Availability | 3.08 | 3.50 | 4.00 | 1.26 | 1.33 | 4.00 |
|  |  | Communication | 2.58 | 2.33 | 2.33 | 0.74 | 2.00 | 3.67 |
|  |  | Knowledge | 3.33 | 3.33 | . | 0.61 | 2.67 | 4.00 |
|  |  | Perseverance | 2.50 | 2.50 | . | 1.04 | 1.33 | 3.67 |
|  |  | Proactive | 2.75 | 2.83 | 3.00 | 0.32 | 2.33 | 3.00 |
|  |  | Support | 3.17 | 3.17 | . | 0.79 | 2.33 | 4.00 |
|  |  | Vision | 2.92 | 2.83 | . | 0.57 | 2.33 | 3.67 |
|  |  | ILS (Total) | 2.90 | 2.79 | . | 0.58 | 2.38 | 3.67 |
| 25 | 7 | Availability | 2.76 | 3.00 | 2.00 | 1.13 | 1.00 | 4.00 |
|  |  | Communication | 2.52 | 2.67 | . | 1.10 | 0.67 | 4.00 |
|  |  | Knowledge | 3.52 | 4.00 | 4.00 | 0.66 | 2.33 | 4.00 |
|  |  | Perseverance | 2.81 | 2.67 | 2.67 | 0.57 | 2.33 | 4.00 |
|  |  | Proactive | 2.86 | 3.00 | 3.00 | 0.74 | 1.67 | 4.00 |
|  |  | Support | 3.00 | 2.67 | 2.67 | 0.61 | 2.33 | 4.00 |
|  |  | Vision | 2.86 | 3.00 | 2.33 | 0.81 | 1.67 | 4.00 |
|  |  | ILS (Total) | 2.90 | 2.76 | . | 0.71 | 1.71 | 4.00 |
| 26 | 9 | Availability | 2.52 | 2.67 | 1.00 | 1.27 | 1.00 | 4.00 |
|  |  | Communication | 2.48 | 2.33 | 2.33 | 0.93 | 1.00 | 4.00 |
|  |  | Knowledge | 3.33 | 3.00 | 3.00 | 0.41 | 3.00 | 4.00 |
|  |  | Perseverance | 3.04 | 3.00 | 3.00 | 0.42 | 2.67 | 4.00 |
|  |  | Proactive | 3.00 | 3.00 | 2.67 | 0.44 | 2.67 | 4.00 |
|  |  | Support | 3.00 | 3.00 | 2.33 | 0.58 | 2.33 | 4.00 |
|  |  | Vision | 2.78 | 3.00 | 3.00 | 0.71 | 1.67 | 4.00 |
|  |  | ILS (Total) | 2.88 | 2.76 | . | 0.56 | 2.24 | 4.00 |
| 27 | 9 | Availability | 3.00 | 3.00 | 3.00 | 0.91 | 1.00 | 4.00 |
|  |  | Communication | 2.81 | 3.00 | 3.00 | 0.53 | 2.00 | 3.67 |
|  |  | Knowledge | 3.04 | 3.00 | 3.00 | 0.70 | 1.67 | 4.00 |
|  |  | Perseverance | 2.78 | 3.00 | 3.00 | 0.58 | 1.67 | 3.33 |
|  |  | Proactive | 2.52 | 2.67 | 2.00 | 0.77 | 1.00 | 3.33 |
|  |  | Support | 3.26 | 3.33 | 3.00 | 0.60 | 2.00 | 4.00 |
|  |  | Vision | 2.89 | 3.00 | 3.00 | 0.47 | 2.00 | 3.33 |
|  |  | ILS (Total) | 2.90 | 2.86 | 2.86 | 0.55 | 1.67 | 3.52 |
| 28 | 8 | Availability | 3.21 | 3.33 | 4.00 | 0.82 | 2.00 | 4.00 |
|  |  | Communication | 3.25 | 3.33 | 2.67 | 0.53 | 2.67 | 4.00 |
|  |  | Knowledge | 3.17 | 3.00 | 3.00 | 0.78 | 2.00 | 4.00 |
|  |  | Perseverance | 3.38 | 3.50 | 3.33 | 0.65 | 2.00 | 4.00 |
|  |  | Proactive | 3.08 | 3.33 | 3.33 | 0.46 | 2.00 | 3.33 |
|  |  | Support | 3.67 | 3.83 | 4.00 | 0.44 | 3.00 | 4.00 |
|  |  | Vision | 3.29 | 3.17 | 3.00 | 0.58 | 2.33 | 4.00 |
|  |  | ILS (Total) | 3.29 | 3.29 | 3.29 | 0.49 | 2.33 | 3.86 |
| 29 | 8 | Availability | 2.79 | 3.00 | 3.00 | 0.64 | 1.67 | 3.67 |
|  |  | Communication | 3.13 | 3.17 | 3.00 | 0.62 | 2.00 | 4.00 |
|  |  | Knowledge | 3.13 | 3.00 | 3.00 | 0.64 | 2.00 | 4.00 |
|  |  | Perseverance | 3.29 | 3.00 | 3.00 | 0.45 | 3.00 | 4.00 |
|  |  | Proactive | 2.75 | 2.67 | 2.67 | 0.46 | 2.00 | 3.33 |
|  |  | Support | 3.46 | 3.33 | 3.00 | 0.47 | 3.00 | 4.00 |
|  |  | Vision | 3.17 | 3.17 | 3.00 | 0.67 | 2.00 | 4.00 |
|  |  | ILS (Total) | 3.10 | 3.00 | 3.00 | 0.47 | 2.52 | 3.81 |
| 30 | 8 | Availability | 2.75 | 2.50 | 2.33 | 0.66 | 2.00 | 4.00 |
|  |  | Communication | 3.04 | 3.00 | 3.00 | 0.21 | 2.67 | 3.33 |
|  |  | Knowledge | 3.21 | 3.00 | 3.00 | 0.73 | 2.00 | 4.00 |
|  |  | Perseverance | 2.88 | 2.83 | 2.67 | 0.56 | 2.00 | 4.00 |
|  |  | Proactive | 2.67 | 2.83 | 3.00 | 0.40 | 2.00 | 3.00 |
|  |  | Support | 3.00 | 2.83 | 2.67 | 0.71 | 2.00 | 4.00 |
|  |  | Vision | 3.29 | 3.17 | 3.00 | 0.38 | 3.00 | 4.00 |
|  |  | ILS (Total) | 2.98 | 2.93 | . | 0.43 | 2.38 | 3.76 |
| 31 | 10 | Availability | 3.40 | 3.83 | 4.00 | 0.75 | 2.00 | 4.00 |
|  |  | Communication | 3.47 | 3.67 | 3.67 | 0.57 | 2.33 | 4.00 |
|  |  | Knowledge | 3.77 | 4.00 | 4.00 | 0.42 | 3.00 | 4.00 |
|  |  | Perseverance | 3.73 | 3.83 | 4.00 | 0.34 | 3.00 | 4.00 |
|  |  | Proactive | 3.67 | 3.83 | 4.00 | 0.44 | 2.67 | 4.00 |
|  |  | Support | 3.83 | 4.00 | 4.00 | 0.42 | 2.67 | 4.00 |
|  |  | Vision | 3.60 | 3.67 | 4.00 | 0.44 | 3.00 | 4.00 |
|  |  | ILS (Total) | 3.64 | 3.69 | 4.00 | 0.41 | 2.67 | 4.00 |
| 32 | 9 | Availability | 3.04 | 3.00 | 3.00 | 0.65 | 2.00 | 4.00 |
|  |  | Communication | 2.78 | 3.00 | 3.00 | 0.65 | 1.67 | 4.00 |
|  |  | Knowledge | 2.81 | 3.00 | 3.00 | 0.71 | 1.67 | 4.00 |
|  |  | Perseverance | 2.78 | 3.00 | 3.00 | 0.82 | 1.67 | 4.00 |
|  |  | Proactive | 2.81 | 3.00 | 3.00 | 0.69 | 1.33 | 4.00 |
|  |  | Support | 3.30 | 3.33 | 3.00 | 0.59 | 2.33 | 4.00 |
|  |  | Vision | 2.89 | 2.67 | 2.67 | 0.58 | 2.00 | 3.67 |
|  |  | ILS (Total) | 2.92 | 2.95 | 2.81 | 0.58 | 1.86 | 3.90 |
| 33 | 8 | Availability | 3.75 | 4.00 | 4.00 | 0.46 | 3.00 | 4.00 |
|  |  | Communication | 3.17 | 3.00 | 3.00 | 0.47 | 2.67 | 4.00 |
|  |  | Knowledge | 3.50 | 3.50 | 3.00 | 0.47 | 3.00 | 4.00 |
|  |  | Perseverance | 3.38 | 3.33 | 3.33 | 0.68 | 2.00 | 4.00 |
|  |  | Proactive | 3.13 | 3.00 | 2.67 | 0.56 | 2.67 | 4.00 |
|  |  | Support | 3.50 | 3.67 | 4.00 | 0.56 | 2.67 | 4.00 |
|  |  | Vision | 3.33 | 3.33 | 2.67 | 0.56 | 2.67 | 4.00 |
|  |  | ILS (Total) | 3.39 | 3.29 | 3.76 | 0.41 | 2.76 | 4.00 |
| 34 | 8 | Availability | 3.79 | 4.00 | 4.00 | 0.31 | 3.33 | 4.00 |
|  |  | Communication | 3.33 | 3.50 | 3.33 | 0.69 | 2.00 | 4.00 |
|  |  | Knowledge | 3.79 | 4.00 | 4.00 | 0.40 | 3.00 | 4.00 |
|  |  | Perseverance | 3.21 | 3.17 | 3.00 | 0.40 | 2.67 | 4.00 |
|  |  | Proactive | 3.42 | 3.50 | 3.00 | 0.50 | 2.67 | 4.00 |
|  |  | Support | 3.71 | 4.00 | 4.00 | 0.45 | 3.00 | 4.00 |
|  |  | Vision | 3.38 | 3.17 | 3.00 | 0.55 | 2.67 | 4.00 |
|  |  | ILS (Total) | 3.52 | 3.50 | . | 0.29 | 3.19 | 4.00 |
| 35 | 12 | Availability | 3.31 | 3.33 | 4.00 | 0.69 | 2.33 | 4.00 |
|  |  | Communication | 3.39 | 3.33 | 4.00 | 0.60 | 2.00 | 4.00 |
|  |  | Knowledge | 3.72 | 4.00 | 4.00 | 0.53 | 2.33 | 4.00 |
|  |  | Perseverance | 3.50 | 3.67 | 4.00 | 0.48 | 2.67 | 4.00 |
|  |  | Proactive | 3.50 | 3.67 | 4.00 | 0.69 | 1.67 | 4.00 |
|  |  | Support | 3.58 | 3.67 | 4.00 | 0.45 | 3.00 | 4.00 |
|  |  | Vision | 3.56 | 3.67 | 4.00 | 0.43 | 2.67 | 4.00 |
|  |  | ILS (Total) | 3.51 | 3.55 | 4.00 | 0.48 | 2.38 | 4.00 |
| 36 | 9 | Availability | 3.30 | 3.00 | 3.00 | 0.48 | 2.67 | 4.00 |
|  |  | Communication | 3.37 | 3.33 | 4.00 | 0.61 | 2.33 | 4.00 |
|  |  | Knowledge | 3.70 | 4.00 | 4.00 | 0.42 | 3.00 | 4.00 |
|  |  | Perseverance | 3.52 | 3.67 | 4.00 | 0.47 | 2.67 | 4.00 |
|  |  | Proactive | 3.19 | 3.00 | 3.00 | 0.58 | 2.33 | 4.00 |
|  |  | Support | 3.67 | 4.00 | 4.00 | 0.41 | 3.00 | 4.00 |
|  |  | Vision | 3.41 | 3.33 | 3.00 | 0.43 | 3.00 | 4.00 |
|  |  | ILS (Total) | 3.45 | 3.57 | 3.05 | 0.38 | 2.90 | 3.90 |
| 37 | 9 | Availability | 2.37 | 2.00 | 1.67 | 1.35 | 0.33 | 4.00 |
|  |  | Communication | 3.07 | 4.00 | 4.00 | 1.16 | 1.00 | 4.00 |
|  |  | Knowledge | 3.44 | 3.33 | 4.00 | 0.55 | 2.67 | 4.00 |
|  |  | Perseverance | 3.04 | 3.33 | 3.33 | 0.77 | 2.00 | 4.00 |
|  |  | Proactive | 3.19 | 3.67 | 3.67 | 0.82 | 1.67 | 4.00 |
|  |  | Support | 3.11 | 3.33 | 3.67 | 0.76 | 1.67 | 4.00 |
|  |  | Vision | 3.22 | 3.67 | 4.00 | 0.87 | 2.00 | 4.00 |
|  |  | ILS (Total) | 3.06 | 3.38 | . | 0.79 | 2.00 | 4.00 |
| 38 | 10 | Availability | 2.37 | 2.50 | 4.00 | 1.38 | 0.67 | 4.00 |
|  |  | Communication | 2.20 | 1.83 | 1.33 | 1.22 | 0.67 | 4.00 |
|  |  | Knowledge | 2.67 | 3.00 | 3.00 | 1.02 | 1.00 | 4.00 |
|  |  | Perseverance | 2.07 | 2.00 | 2.00 | 1.02 | 0.67 | 4.00 |
|  |  | Proactive | 2.20 | 2.00 | 2.00 | 1.00 | 0.67 | 4.00 |
|  |  | Support | 2.80 | 2.67 | 2.67 | 1.02 | 1.33 | 4.00 |
|  |  | Vision | 2.43 | 3.00 | 3.00 | 1.35 | 0.33 | 4.00 |
|  |  | ILS (Total) | 2.39 | 2.38 | . | 1.08 | 0.86 | 4.00 |
| 39 | 9 | Availability | 3.04 | 3.00 | 4.00 | 1.05 | 1.33 | 4.00 |
|  |  | Communication | 2.93 | 3.00 | 2.67 | 0.98 | 1.00 | 4.00 |
|  |  | Knowledge | 3.11 | 3.00 | 3.00 | 0.75 | 2.00 | 4.00 |
|  |  | Perseverance | 3.07 | 3.00 | 4.00 | 0.76 | 2.00 | 4.00 |
|  |  | Proactive | 2.70 | 2.67 | 2.00 | 0.84 | 1.67 | 4.00 |
|  |  | Support | 3.30 | 3.67 | 4.00 | 0.81 | 2.00 | 4.00 |
|  |  | Vision | 3.22 | 3.33 | 4.00 | 0.87 | 1.33 | 4.00 |
|  |  | ILS (Total) | 3.05 | 3.10 | . | 0.73 | 1.67 | 4.00 |
| 40 | 8 | Availability | 0.71 | 0.67 | 0.00 | 0.74 | 0.00 | 2.00 |
|  |  | Communication | 1.00 | 1.00 | 0.00 | 0.80 | 0.00 | 2.33 |
|  |  | Knowledge | 1.17 | 1.33 | 2.00 | 0.85 | 0.00 | 2.00 |
|  |  | Perseverance | 0.88 | 0.83 | 0.00 | 0.75 | 0.00 | 2.33 |
|  |  | Proactive | 1.00 | 1.17 | 1.67 | 0.71 | 0.00 | 1.67 |
|  |  | Support | 0.88 | 1.00 | 0.00 | 0.62 | 0.00 | 1.67 |
|  |  | Vision | 1.08 | 1.17 | 0.00 | 0.94 | 0.00 | 2.67 |
|  |  | ILS (Total) | 0.96 | 1.05 | 0.95 | 0.66 | 0.00 | 1.90 |
| 41 | 11 | Availability | 3.18 | 3.33 | 4.00 | 0.72 | 2.00 | 4.00 |
|  |  | Communication | 3.33 | 3.33 | 3.00 | 0.61 | 2.00 | 4.00 |
|  |  | Knowledge | 3.33 | 3.00 | 3.00 | 0.56 | 2.67 | 4.00 |
|  |  | Perseverance | 3.00 | 3.00 | 3.00 | 0.61 | 2.00 | 4.00 |
|  |  | Proactive | 2.82 | 2.67 | 2.67 | 0.50 | 2.00 | 4.00 |
|  |  | Support | 3.33 | 3.33 | 3.00 | 0.61 | 2.00 | 4.00 |
|  |  | Vision | 3.15 | 3.33 | 3.33 | 0.52 | 2.00 | 4.00 |
|  |  | ILS (Total) | 3.16 | 3.05 | 2.95 | 0.51 | 2.10 | 4.00 |
| 42 | 10 | Availability | 3.47 | 3.83 | 4.00 | 0.69 | 2.00 | 4.00 |
|  |  | Communication | 3.63 | 3.83 | 4.00 | 0.46 | 3.00 | 4.00 |
|  |  | Knowledge | 3.73 | 4.00 | 4.00 | 0.44 | 3.00 | 4.00 |
|  |  | Perseverance | 3.40 | 3.50 | 4.00 | 0.66 | 2.33 | 4.00 |
|  |  | Proactive | 3.47 | 3.50 | 4.00 | 0.57 | 2.33 | 4.00 |
|  |  | Support | 3.63 | 4.00 | 4.00 | 0.48 | 3.00 | 4.00 |
|  |  | Vision | 3.63 | 4.00 | 4.00 | 0.48 | 3.00 | 4.00 |
|  |  | ILS (Total) | 3.57 | 3.81 | 4.00 | 0.50 | 2.71 | 4.00 |
| 43 | 7 | Availability | 0.86 | 0.67 | 0.00 | 0.86 | 0.00 | 2.00 |
|  |  | Communication | 0.67 | 0.33 | 0.00 | 0.92 | 0.00 | 2.00 |
|  |  | Knowledge | 0.95 | 1.00 | 0.33 | 0.80 | 0.00 | 2.00 |
|  |  | Perseverance | 0.71 | 0.33 | 0.00 | 0.91 | 0.00 | 2.00 |
|  |  | Proactive | 0.76 | 0.33 | 0.00 | 0.88 | 0.00 | 2.00 |
|  |  | Support | 0.86 | 0.33 | 0.00 | 0.90 | 0.00 | 2.00 |
|  |  | Vision | 0.86 | 1.00 | 0.00 | 0.90 | 0.00 | 2.00 |
|  |  | ILS (Total) | 0.81 | 0.62 | 0.14 | 0.85 | 0.10 | 2.00 |
| 44 | 4 | Availability | 2.25 | 2.00 | 1.00 | 1.50 | 1.00 | 4.00 |
|  |  | Communication | 1.33 | 1.00 | 1.00 | 0.90 | 0.67 | 2.67 |
|  |  | Knowledge | 1.83 | 1.67 | 1.67 | 0.84 | 1.00 | 3.00 |
|  |  | Perseverance | 1.50 | 1.17 | 1.00 | 0.79 | 1.00 | 2.67 |
|  |  | Proactive | 1.50 | 1.33 | 1.00 | 0.64 | 1.00 | 2.33 |
|  |  | Support | 1.50 | 1.17 | 1.00 | 0.79 | 1.00 | 2.67 |
|  |  | Vision | 1.33 | 1.17 | . | 0.72 | 0.67 | 2.33 |
|  |  | ILS (Total) | 1.61 | 1.31 | . | 0.84 | 1.00 | 2.81 |
| 45 | 12 | Availability | 2.36 | 2.67 | 3.00 | 1.35 | 0.00 | 4.00 |
|  |  | Communication | 2.72 | 3.00 | 2.00 | 1.11 | 0.00 | 4.00 |
|  |  | Knowledge | 2.67 | 2.83 | 3.00 | 0.68 | 1.67 | 4.00 |
|  |  | Perseverance | 2.53 | 3.00 | 3.00 | 1.06 | 0.00 | 4.00 |
|  |  | Proactive | 2.58 | 2.83 | 2.00 | 1.02 | 0.00 | 4.00 |
|  |  | Support | 2.69 | 2.67 | 2.67 | 0.90 | 1.00 | 4.00 |
|  |  | Vision | 2.61 | 3.00 | 3.00 | 1.13 | 0.00 | 4.00 |
|  |  | ILS (Total) | 2.60 | 2.90 | 2.05 | 0.98 | 0.38 | 3.76 |
| 46 | 4 | Availability | 2.08 | 2.00 | 2.00 | 1.23 | 0.67 | 3.67 |
|  |  | Communication | 1.58 | 2.00 | 2.33 | 1.10 | 0.00 | 2.33 |
|  |  | Knowledge | 1.83 | 2.00 | 2.00 | 0.58 | 1.00 | 2.33 |
|  |  | Perseverance | 1.58 | 1.67 | . | 0.74 | 0.67 | 2.33 |
|  |  | Proactive | 1.67 | 1.67 | 1.67 | 1.09 | 0.33 | 3.00 |
|  |  | Support | 2.42 | 2.50 | . | 1.42 | 0.67 | 4.00 |
|  |  | Vision | 1.67 | 2.00 | 2.00 | 0.67 | 0.67 | 2.00 |
|  |  | ILS (Total) | 1.83 | 2.00 | . | 0.92 | 0.57 | 2.76 |
| 47 | 7 | Availability | 2.33 | 2.00 | 1.00 | 1.19 | 1.00 | 4.00 |
|  |  | Communication | 2.29 | 2.33 | 1.33 | 0.97 | 1.33 | 3.67 |
|  |  | Knowledge | 2.38 | 2.67 | 3.00 | 0.71 | 1.33 | 3.00 |
|  |  | Perseverance | 2.19 | 2.00 | 1.00 | 1.05 | 1.00 | 3.67 |
|  |  | Proactive | 2.05 | 2.00 | 1.67 | 0.83 | 0.67 | 3.00 |
|  |  | Support | 2.81 | 3.00 | 2.00 | 0.81 | 2.00 | 4.00 |
|  |  | Vision | 1.86 | 1.67 | 1.00 | 1.02 | 0.67 | 3.00 |
|  |  | ILS (Total) | 2.27 | 2.14 | . | 0.87 | 1.14 | 3.29 |
| 48 | 4 | Availability | 3.25 | 3.00 | 3.00 | 0.50 | 3.00 | 4.00 |
|  |  | Communication | 3.33 | 3.17 | 3.00 | 0.47 | 3.00 | 4.00 |
|  |  | Knowledge | 3.58 | 3.67 | 4.00 | 0.50 | 3.00 | 4.00 |
|  |  | Perseverance | 3.33 | 3.33 | . | 0.61 | 2.67 | 4.00 |
|  |  | Proactive | 3.42 | 3.50 | 4.00 | 0.69 | 2.67 | 4.00 |
|  |  | Support | 3.08 | 3.50 | 4.00 | 1.26 | 1.33 | 4.00 |
|  |  | Vision | 3.25 | 3.00 | 3.00 | 0.50 | 3.00 | 4.00 |
|  |  | ILS (Total) | 3.32 | 3.26 | . | 0.55 | 2.76 | 4.00 |
| 49 | 4 | Availability | 3.75 | 4.00 | 4.00 | 0.50 | 3.00 | 4.00 |
|  |  | Communication | 3.75 | 3.67 | 3.67 | 0.17 | 3.67 | 4.00 |
|  |  | Knowledge | 3.92 | 4.00 | 4.00 | 0.17 | 3.67 | 4.00 |
|  |  | Perseverance | 3.75 | 4.00 | 4.00 | 0.50 | 3.00 | 4.00 |
|  |  | Proactive | 3.33 | 3.33 | . | 0.61 | 2.67 | 4.00 |
|  |  | Support | 3.67 | 3.67 | 3.33 | 0.38 | 3.33 | 4.00 |
|  |  | Vision | 3.83 | 3.83 | 3.67 | 0.19 | 3.67 | 4.00 |
|  |  | ILS (Total) | 3.71 | 3.74 | . | 0.27 | 3.38 | 4.00 |
| 50 | 8 | Availability | 3.63 | 4.00 | 4.00 | 0.52 | 3.00 | 4.00 |
|  |  | Communication | 3.67 | 4.00 | 4.00 | 0.47 | 3.00 | 4.00 |
|  |  | Knowledge | 3.67 | 4.00 | 4.00 | 0.47 | 3.00 | 4.00 |
|  |  | Perseverance | 3.71 | 4.00 | 4.00 | 0.45 | 3.00 | 4.00 |
|  |  | Proactive | 3.63 | 4.00 | 4.00 | 0.52 | 3.00 | 4.00 |
|  |  | Support | 3.67 | 4.00 | 4.00 | 0.47 | 3.00 | 4.00 |
|  |  | Vision | 3.67 | 4.00 | 4.00 | 0.47 | 3.00 | 4.00 |
|  |  | ILS (Total) | 3.66 | 4.00 | 4.00 | 0.48 | 3.00 | 4.00 |
| 51 | 4 | Availability | 3.50 | 4.00 | 4.00 | 1.00 | 2.00 | 4.00 |
|  |  | Communication | 3.67 | 3.83 | 4.00 | 0.47 | 3.00 | 4.00 |
|  |  | Knowledge | 3.75 | 4.00 | 4.00 | 0.50 | 3.00 | 4.00 |
|  |  | Perseverance | 3.58 | 3.83 | 4.00 | 0.63 | 2.67 | 4.00 |
|  |  | Proactive | 3.42 | 3.83 | 4.00 | 0.96 | 2.00 | 4.00 |
|  |  | Support | 3.67 | 3.83 | 4.00 | 0.47 | 3.00 | 4.00 |
|  |  | Vision | 3.75 | 3.83 | 4.00 | 0.32 | 3.33 | 4.00 |
|  |  | ILS (Total) | 3.62 | 3.88 | 4.00 | 0.61 | 2.71 | 4.00 |
| 52 | 10 | Availability | 3.63 | 4.00 | 4.00 | 0.58 | 2.33 | 4.00 |
|  |  | Communication | 3.27 | 3.50 | 4.00 | 0.83 | 2.00 | 4.00 |
|  |  | Knowledge | 3.53 | 4.00 | 4.00 | 0.69 | 2.00 | 4.00 |
|  |  | Perseverance | 3.10 | 3.00 | 3.00 | 0.74 | 2.00 | 4.00 |
|  |  | Proactive | 3.53 | 3.83 | 4.00 | 0.59 | 2.33 | 4.00 |
|  |  | Support | 3.67 | 4.00 | 4.00 | 0.50 | 2.67 | 4.00 |
|  |  | Vision | 3.53 | 4.00 | 4.00 | 0.72 | 2.00 | 4.00 |
|  |  | ILS (Total) | 3.47 | 3.64 | 4.00 | 0.59 | 2.24 | 4.00 |

*Note.* Std. Dev. = standard deviation.
